# Positioning the Model Bacterial Organelle, the Carboxysome

**DOI:** 10.1128/mBio.02519-19

**Published:** 2021-05-11

**Authors:** Joshua S. MacCready, Anthony G. Vecchiarelli

**Affiliations:** a Department of Molecular, Cellular, and Developmental Biology, University of Michigan, Ann Arbor, Michigan, USA; University of Texas Health Science Center at Houston

**Keywords:** bacterial microcompartments, ParA ATPase, McdA, McdB, subcellular organization

## Abstract

Bacterial microcompartments (BMCs) confine a diverse array of metabolic reactions within a selectively permeable protein shell, allowing for specialized biochemistry that would be less efficient or altogether impossible without compartmentalization. BMCs play critical roles in carbon fixation, carbon source utilization, and pathogenesis. Despite their prevalence and importance in bacterial metabolism, little is known about BMC “homeostasis,” a term we use here to encompass BMC assembly, composition, size, copy-number, maintenance, turnover, positioning, and ultimately, function in the cell. The carbon-fixing carboxysome is one of the most well-studied BMCs with regard to mechanisms of self-assembly and subcellular organization. In this minireview, we focus on the only known BMC positioning system to date—the maintenance of carboxysome distribution (Mcd) system, which spatially organizes carboxysomes. We describe the two-component McdAB system and its proposed diffusion-ratchet mechanism for carboxysome positioning. We then discuss the prevalence of McdAB systems among carboxysome-containing bacteria and highlight recent evidence suggesting how liquid-liquid phase separation (LLPS) may play critical roles in carboxysome homeostasis. We end with an outline of future work on the carboxysome distribution system and a perspective on how other BMCs may be spatially regulated. We anticipate that a deeper understanding of BMC organization, including nontraditional homeostasis mechanisms involving LLPS and ATP-driven organization, is on the horizon.

## INTRODUCTION

Compartmentalization of specialized processes is a fundamental feature across all domains of life. Often referred to as organelles, these structures are classified as either membrane-bound, possessing a semipermeable membrane comprising lipid and protein, or membraneless (phase defined), also referred to as biomolecular condensates (1). Historically, the term “organelle” has been strictly attributed to eukaryotic organisms; however, we now know that bacteria also possess such structures, including anammoxosomes (2, 3), magnetosomes (4, 5), chromatophores (6, 7), chlorosomes (8), bacterial microcompartments (BMCs) (9), and nanocompartments (also referred to as encapsulins) (10, 11). It is also becoming evident that bacteria have a variety of membraneless organelles involved in diverse biological processes that form via liquid-liquid phase separation, or LLPS (12, 13). Among these bacterial organelles, the carbon-fixing BMC called the carboxysome is one of the most well studied to date, particularly with regard to mechanisms of self-assembly and subcellular organization.

Enzyme compartmentalization spatially organizes metabolic reactions and increases efficiency. BMCs confine a diverse array of sensitive anabolic or catabolic reactions by encapsulating key enzymes within a selectively permeable protein shell (9). This method of compartmentalization can locally increase the concentration of enzymes and substrates (14, 15), prevent leakage of toxic intermediates (16), and create microenvironments distinct from conditions in the cytoplasm, such as pH, redox state, and cofactor pools (17). In short, BMCs allow for specialized biochemistry that would be less efficient or altogether impossible without compartmentalization.

The genes that encode BMC shell proteins and enzymes are genomically clustered and organized in coregulated operons (16, 18). The mining of sequenced bacterial genomes has revealed 23 different BMC types in 29 bacterial phyla, including those found in the human gut microbiome (18). Certain BMCs have been shown to play critical roles in pathogenesis and human health (19, 20), yet among the variety of BMC types identified, relatively few have garnered experimental support for their proposed functions in the cell and have been divided metabolically into anabolic carboxysomes and catabolic metabolosomes. Metabolosomes that have been experimentally characterized utilize propanediol (PDU) (21–23), ethanolamine (EUT) (24), fucose and rhamnose (25, 26), 1-amino-2-propanol (27, 28), and choline (29, 30). Despite their functional diversity, all metabolosomes create, sequester, and detoxify volatile aldehyde intermediates that can kill the cell (26, 31–34). In 2010, a Ras-like GTPase called PduV was suggested to play a role in positioning the PDU BMC via an unknown filament-based mechanism (35). Aside from this single study, it remains unclear how metabolosomes are spatially organized in bacterial cells.

Carboxysomes encapsulate the most abundant enzyme on Earth (36), ribulose-1,5-bisphosphate carboxylase/oxygenase (Rubisco), and represent the best-studied model for understanding BMC biology, specifically, assembly and organization in the cell. The carboxysome shell, together with the coencapsulated enzyme carbonic anhydrase that converts bicarbonate to CO_2_ (the substrate for Rubisco), increases the local concentration of CO_2_, which enhances the efficiency and selectivity of Rubisco (37). Along with its eukaryotic equivalent in algae, the pyrenoid, these protein-based organelles are responsible for roughly half of global carbon fixation (38, 39).

Since BMCs play critical roles in carbon fixation, carbon source utilization, and pathogenesis, their functions are of great ecological, biotechnological, and medical interest. Despite their importance, little is known about the mechanisms used by bacteria to regulate BMC “homeostasis,” a term we use here to encompass the dynamic equilibrium among the interrelated aspects of BMC assembly, composition, size, copy number, maintenance, turnover, positioning, and ultimately, function in the cell. In this minireview, we focus on the only known BMC positioning system to date—the maintenance of carboxysome distribution (Mcd) system, which spatially organizes carboxysomes (40, 41). We start with a general overview of carboxysome biology, as this is the focus of a number of excellent recent reviews (42, 43). Second, we introduce the two-component McdAB system and summarize its proposed diffusion-ratchet mechanism of carboxysome positioning (40). We then highlight recent work showing that McdAB systems are widespread among carboxysome-containing bacteria (41, 44). We also discuss recent findings that suggest how LLPS may play key roles in carboxysome homeostasis (12, 43–47). Finally, we end with an outline of future work on carboxysome positioning by the McdAB system and a perspective on how other BMCs may also be spatially regulated in the cell.

## CARBOXYSOMES—THE MODEL BMC

**FIG 1:**
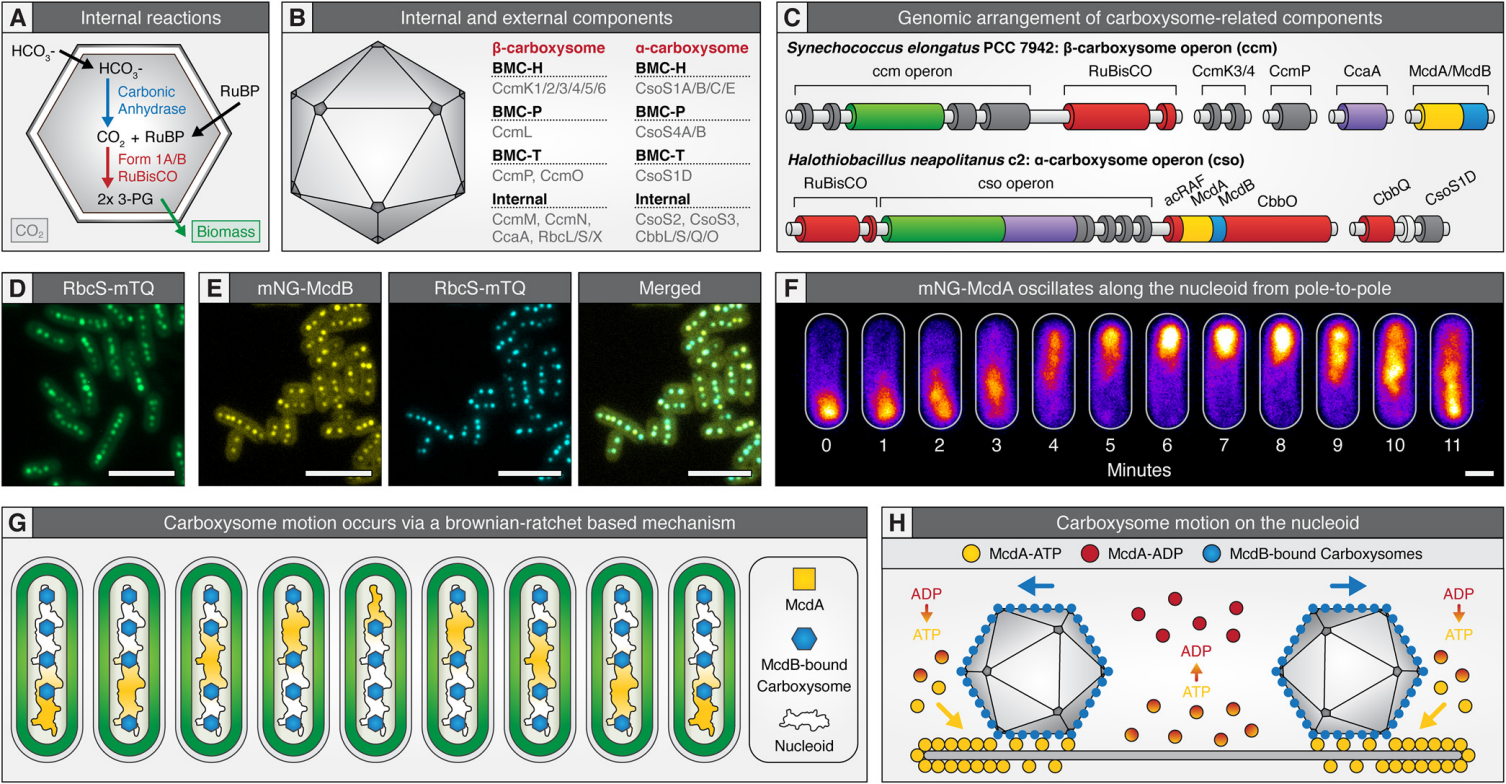
Carboxysomes are spatially organized by the McdAB system. (A) Cartoon illustration of internal carboxysome reactions among α- and β-carboxysomes. (B) Cartoon illustrations of α- and β-carboxysome components. (C) Genomic arrangement of model α- and β-carboxysome operons. Dark gray, shell component; red, Rubisco-related component; green, Rubisco aggregating component; purple, carbonic anhydrase; yellow, McdA; blue, McdB. (D) Visualization of carboxysome arrangement in *S. elongatus* using a fluorescent fusion of the small subunit of Rubisco, RbcS-mTQ. Scale bar = 5 μm. (E) McdB colocalizes with carboxysomes in *S. elongatus*. Scale bar = 5 μm. (F) McdA oscillates from pole to pole in *S. elongatus*. Scale bar = 1 μm. (G) Model for gross carboxysome motion via McdA gradients on the cyanobacterial nucleoid. (H) Model for individual carboxysome motions via a burnt-bridge Brownian ratchet mechanism. Data for panels D to F are from reference 40 (https://creativecommons.org/publicdomain/zero/1.0/).

Carboxysomes are essential bacterial organelles, commonly described as icosahedral in shape, that compartmentalize the oxygen-sensitive process of carbon fixation using a semipermeable protein shell (9, 17, 48). Specifically, since CO_2_ and O_2_ compete for binding Rubisco, the coencapsulation of Rubisco and carbonic anhydrase within a selectively permeable protein shell generates a high internal CO_2_ environment that drives Rubisco reactions toward the Calvin-Benson-Bassham cycle (CO_2_ substrate) and away from the process of photorespiration (O_2_ substrate) (Fig. 1A) (49–52). Through this mechanism, carboxysomes contribute to greater than 35% of global carbon fixation through atmospheric CO_2_ assimilation (39).

To date, two types of carboxysomes have been characterized, α and β. α-carboxysomes encapsulate form 1A Rubisco, and β-carboxysomes encapsulate form 1B Rubisco. Despite this difference, α- and β-carboxysomes have similar Rubisco kinetics (53). All cyanobacteria possess either α- or β-carboxysomes, whereas several carbon-fixing proteobacteria and some actinobacteria only possess α-carboxysomes. It is believed that α-carboxysomes emerged in proteobacteria and were subsequently horizontally transferred to cyanobacteria early in their evolution, thus creating the two distinct lineages of cyanobacteria—α-cyanobacteria possessing α-carboxysomes and β-cyanobacteria possessing β-carboxysomes (54).

While α- and β-carboxysomes possess similar functions, they are composed of structurally and phyletically distinct protein components (Fig. 1B) (54). The vast majority of α- and β-carboxysome-related genes tend to form operons with their respective encapsulated enzymes (Fig. 1C) (18). Indeed, in the model β-cyanobacterium Synechococcus elongatus PCC 7942 (here, *S. elongatus*) the core *ccm* operon (carbon concentrating mechanism) is genomically located next to the genes encoding Rubisco, *rbcL* and *rbcS* (Pfam accession entries PF02788/PF00016 and PF00101). The first component of the *ccm* operon is the hexameric shell protein CcmK2 (PF00936), which laterally assembles to form the faces of the icosahedron (55, 56). The next component, pentameric shell protein CcmL (PF03319), caps the icosahedral vertices (57–59). Next, the internal carboxysome component CcmM (PF00132/PF14602/PF00101), which is expressed as a long (58 kDa) and short form (35 kDa), aggregates Rubisco to form a “procarboxysome,” and this complex is connected to the CcmK2 shell via the protein CcmN (PF00132) (60–63). The last component in the *ccm* operon is the tandem shell protein CcmO (PF00936), which has been hypothesized to function as a “zipper” that connects the edges of the CcmK2 faces (56). The remaining carboxysome components are all distantly located from the *ccm* operon. The minor hexameric shell proteins, CcmK3 and CcmK4 (PF00936), form heterohexamers and are believed to modulate carboxysome permeability to increase or decrease metabolite shuffling with the cytoplasm (64). Carbonic anhydrase, CcaA (PF00484), is recruited to the procarboxysome by CcmM, positioned in close proximity to Rubisco, and encapsulated (65–67). Lastly, the pseudohexameric shell protein CcmP (PF00936) contains a large central pore that opens and closes in response to ligand binding (68, 69).

The chemoautotrophic proteobacterium Halothiobacillus neapolitanus c2 (here, *H. neapolitanus*) is the model organism for the study of α-carboxysomes. The core α-carboxysome *cso* operon (CarboxySOme) is much more highly conserved in structure than the *ccm* operon and is also genomically located next to the genes encoding the large and small subunits of Rubisco, *cbbL* and *cbbS* (PF02788/PF00016 and PF00101) (Fig. 1C). The *cso* operon significantly differs from the *ccm* operon in several ways. First, while the proteins CcmM and CcmN are required to aggregate Rubisco and carbonic anhydrase into a procarboxysome and tether this complex to the shell of β-carboxysomes, the first gene in the *cso* operon, *csoS2* (PF12288), solely fulfills these roles in α-carboxysomes and is almost always genomically followed by carbonic anhydrase, *csoS3* (70) (Fig. 1B). Next, the pentameric paralog shell proteins, CsoS4A (PF03319) and CsoS4B (PF03319), are thought to function similarly to β-carboxysome CcmL, capping the vertices (71). The last components of the *cso* operon, the hexameric shell proteins CsoS1A (PF00936), CsoS1B, and CsoS1C, end the traditional *cso* operon and form the face of the icosahedral shape, a function similar to β-carboxysome component CcmK2, and also interact with the core Rubisco-aggregating component CsoS2 (47, 70, 72–74). Remaining α-carboxysome components are typically located outside the *cso* operon but still remain in close genomic proximity. Among these components, the double-stacking trimeric shell protein CsoS1D (PF00936) is usually found downstream of the *cso* operon and possesses gated pores analogous to β-carboxysome component CcmP (68, 73, 75). Lastly, although not present in *H. neapolitanus*, the hexameric shell protein CsoS1E (PF00936) often precedes the *cso* operon in α-cyanobacteria adapted to low light (73).

A full understanding of the protein interactome and internal organization of both carboxysome types is within reach, but given the number of self-assembling components, why do carboxysomes, or BMCs in general, not aggregate in the cell? As we highlight in the next section, the study of carboxysome organization and its recently identified anti-aggregation system is in its infancy.

## THE MCDAB SYSTEM POSITIONS α- AND β-CARBOXYSOMES

The dynamic assembly and spatial organization of β-carboxysomes has been visualized in living *S. elongatus* cells using fluorescent fusions to internal or external carboxysome components (40, 63, 76–79). β-carboxysomes are uniformly positioned along the longitudinal cell axis (40, 76) (Fig. 1D). This positioning, along with carboxysome composition, diameter, and mobility, is dynamically and sensitively regulated by changes in temperature, CO_2_ levels, light intensity, and wavelength during cell growth (78–81). How these external cues regulate β-carboxysome homeostasis remains unknown.

For α-carboxysomes, *in vivo* imaging has mostly been restricted to electron micrographs of *H. neapolitanus* cells (54, 82–85). In general, *H. neapolitanus* α-carboxysomes are greater in number (4 to 18) and smaller (40- to 200-nm diameter) compared to the fewer (3 to 5) and larger (90 to 600 nm) β-carboxysomes of *S. elongatus* (40, 54, 76, 78, 84, 86). Despite these differences, *H. neapolitanus* α-carboxysomes are also distributed down the cell length (14, 41, 87).

It was recently revealed that a ParA family ATPase, termed maintenance of carboxysome distribution protein A (McdA), is required for spatially organizing both α- (41) and β-carboxysomes (40, 76). All members of this broad ParA-family of ATPases encode a deviant Walker A box as an ATP-binding motif, and the members that have been primarily studied are those involved in segregating and positioning genetic cargos, such as chromosomes and plasmids (88, 89). However, a growing list of ParA family members have been implicated in positioning functionally diverse protein complexes, including those involved in secretion (90, 91), chemotaxis (92–94), conjugation (95), cell division (96, 97), and cell motility (98, 99). Thus, these ParA family ATPases are critical for shaping and maintaining the internal architecture of bacterial cells for a number of biological processes. McdA is the first ParA family ATPase shown to be responsible for spatially organizing a metabolic process, specifically, carbon fixation.

ParA family ATPases require a partner protein for positioning their cognate intracellular cargo. The partner protein is usually encoded immediately downstream of the *parA* gene and in the same operon. For genetic cargos, the ParA partner protein is called ParB. ParB proteins bind specifically to DNA-binding sites on a chromosome or plasmid, thus demarcating the genetic element as “cargo” for positioning by the cognate ParA. Consistently, a small partner protein expressed downstream of the *mcdA* gene, and in the same operon, was recently discovered called McdB (40, 41). McdB proteins strongly colocalize with β-carboxysomes in *S. elongatus* (Fig. 1E) and α-carboxysomes in *H. neapolitanus*, and both are required for carboxysome positioning in their respective organisms.

A mechanistic understanding of the two-component McdAB system has largely come from the study of β-carboxysome positioning in *S. elongatus* (40). McdB associates with β-carboxysomes through multiple shell protein interactions. While McdB associates with carboxysomes, McdA dimerizes in the presence of ATP and binds the nucleoid via nonspecific DNA interactions (40, 100). McdB stimulates the ATPase activity of McdA as well as McdA release from a nonspecific DNA substrate *in vitro*, which translates *in vivo* to McdA release from the nucleoid in the vicinity of McdB-bound carboxysomes (40). The interaction between McdB-bound carboxysomes and nucleoid-localized McdA causes (i) an McdA depletion zone to form on the nucleoid in the vicinity of carboxysomes, (ii) a global break in McdA symmetry along the nucleoid, (iii) a movement of carboxysomes toward increased McdA concentrations, and (iv) a pole-to-pole oscillation of McdA that emerges across space and time (Fig. 1F). This sequence of events is very similar to what is observed with ParA-based plasmid partitioning, where the mechanism has been described as a Brownian ratchet (Fig. 1G) (101–108). It was recently shown through mathematical modeling that this Brownian-ratchet mechanism can also account for the active distribution of McdB-bound carboxysomes responding to dynamic McdA concentration gradients on the nucleoid (Fig. 1H) (40).

In the absence of either McdA or McdB, α- and β-carboxysomes still self-assemble but form aggregates that largely mislocalize toward polar regions of the cell (40, 41). Interestingly, the degradation of inactive carboxysomes was recently found to also occur near polar regions of a cyanobacterial cell (109). It remains to be determined how carboxysome mispositioning and aggregation, due to the lack of a functional McdAB system, influences carboxysome turnover and function.

α- and β-carboxysome aggregation does not result in a high CO_2_-requiring phenotype (40, 110), which suggests McdAB systems are not crucial for growth under the optimal growth conditions typically used in a lab setting. However, McdAB deletion strains of *S. elongatus* have recently been shown to display slower growth rates, cell elongation, asymmetric cell division, and altered cellular levels of Rubisco (80). Deletion of McdB elicited stronger mutant phenotypes compared to the deletion of McdA, which suggests that McdB plays a critical, but currently unknown, role in the carbon-fixing function of carboxysomes, outside of its role in positioning with McdA.

## MCDAB SYSTEMS ARE WIDESPREAD AMONG CARBOXYSOME-CONTAINING BACTERIA

**FIG 2:**
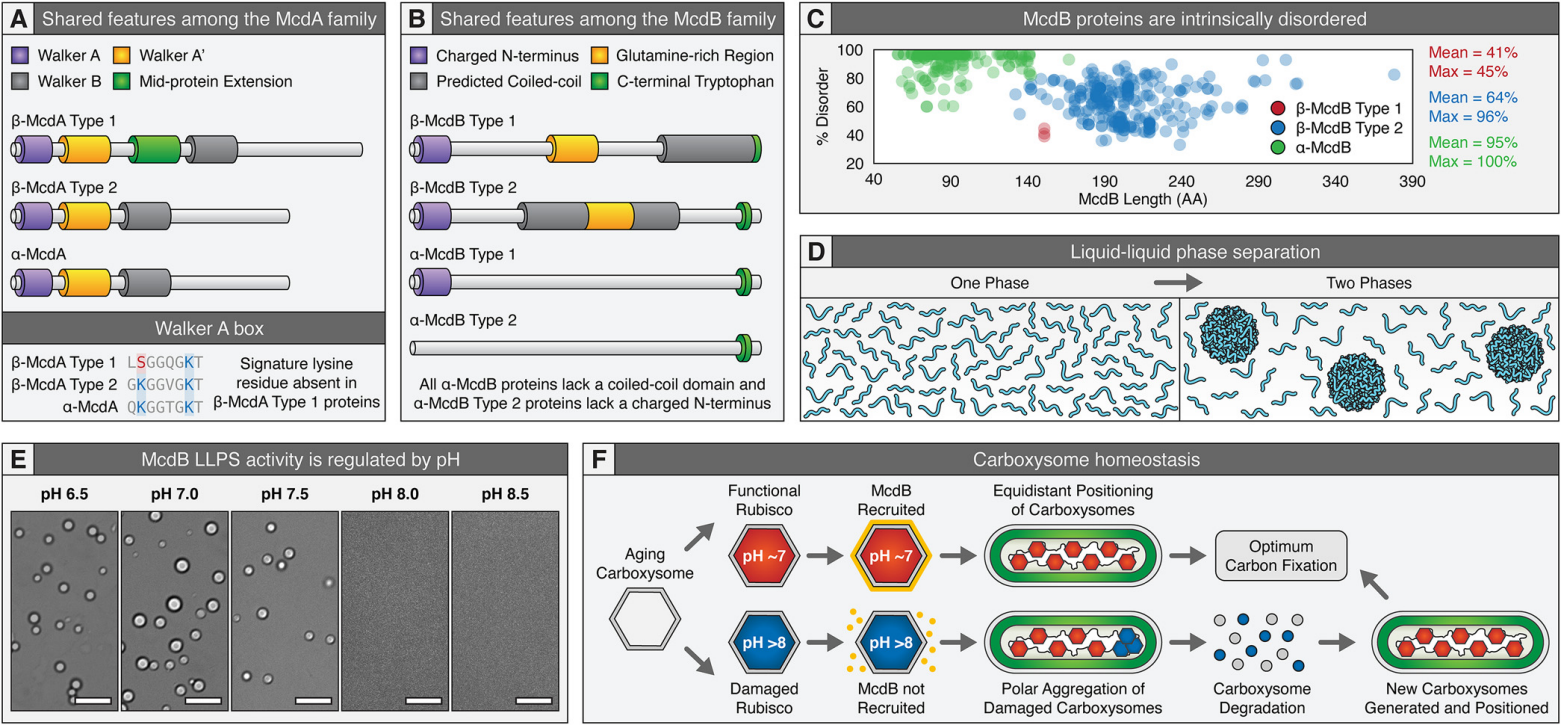
Conserved features and functions of McdAB systems in bacteria. (A) Known amino acid sequence features of the McdA family of proteins. Alignment of Walker A residues (bottom) highlighting the absence of the signature lysine residue in β-McdA type 1 proteins. (B) Known amino acid sequence features of the McdB family of proteins. (C) PONDR disorder scatterplots for all McdB protein types. β-McdB type 1 proteins (red) are on average 41% disordered, β-McdB type 2 proteins (blue) are on average 64% disordered, and α-McdB proteins (green) are significantly more disordered at ∼95%. This dramatic difference in disorder for α-McdB proteins is likely due to the lack of the predicted coiled-coil found in both β-McdB types. (D) Cartoon illustrating liquid-liquid phase separation. (E) Differential interference contrast (DIC) images showing that purified *S. elongatus* McdB has pH-dependent LLPS activity *in vitro*. McdB forms liquid-like droplets at a pH of ≤7.5 but remains soluble at a pH of ≥8. Scale bar = 10 μm. Data are from reference 44. (F) Proposed model for carboxysome homeostasis by the McdAB system. Metabolically active carboxysomes (red) have a lower intrashellular pH (∼7) compared to the cytoplasm of light-acclimated *S. elongatus* (pH > 8). The lower pH of active carboxysomes recruits McdB via its pH-dependent LLPS activity. McdB-bound carboxysomes are recognized by McdA and distributed on the nucleoid. Metabolically inactive carboxysomes (blue), on the other hand, would have the same basic pH as the cytoplasm. McdB does not under LLPS at this pH and would therefore not be recruited to inactive carboxysomes. Without McdB, inactive carboxysomes would not be recognized and distributed by McdA on the nucleoid. As a result, inactive carboxysomes become nucleoid excluded to the cell poles, where they are degraded, and the components are recycled.

To date, the McdAB system has only been experimentally shown as essential for carboxysome positioning in *S. elongatus* and *H. neapolitanus* (40, 41). Bioinformatics have shown that, while absent in α-cyanobacteria, McdAB systems are widespread among β-cyanobacteria and α-carboxysome-containing proteobacteria (41, 44). The McdA and McdB proteins identified have an incredible amount of diversity across these two phyla and thus have been classified based on shared amino acid sequence features. There are currently four distinct types of McdAB systems, two in cyanobacteria (β-McdAB type 1 and 2) and two in proteobacteria (α-McdAB type 1 and 2). Among β-cyanobacteria such as *S. elongatus*, β-McdAB type 1 systems possess an McdA protein that lacks the signature lysine residue in the deviant Walker A box, a sequence feature that defines the ParA family of ATPases (Fig. 2A). β-McdA type 1 proteins also possess a large midprotein extension of unknown function. Alternatively, β-McdAB type 2 systems, which are more ancestral in cyanobacteria than type 1, possess an McdA protein that contains the signature lysine residue of the ParA family and lacks the large midprotein extension. β-McdB type 1 proteins possess a central glutamine-rich region and a predicted C-terminal coiled-coil domain (Fig. 2B). β-McdB type 2 proteins also possess a central glutamine-rich region and a predicted coiled coil, but it is predicted to be centrally located.

Among the α-McdAB systems of carboxysome-containing proteobacteria, all α-McdB proteins lack the coiled-coil domain found in β-McdB proteins (Fig. 2B). We recently found that *S. elongatus* β-McdB type 1 forms a hexamer, *Synechococcus* sp. strain PCC 7002 β-McdB type 2 forms a dimer, and consistent with lacking a predicted coiled-coil, *H. neapolitanus* α-McdB is a monomer. Therefore, the predicted coiled coil exclusive to β-McdB proteins is likely required for oligomerization and is important for β-carboxysome positioning and function, whereas α-McdB proteins function as monomers.

α-McdA proteins do not possess any distinguishing features from β-McdA type 2 proteins (Fig. 2A). The delineation between the two α-McdAB system types is solely based on an additional genomic copy of the α-McdB protein that has a unique sequence feature—the lack of a charged N terminus found in all other McdB types (Fig. 2B). α-*mcdB* type 1 genes are always genomically located downstream of the α-*mcdA* gene and can be found either within or distant from the *cso* carboxysome operon. In cases where α-*mcdAB* is distant from the *cso* operon, a second α-*mcdB* gene (termed α-McdB type 2) is sometimes present in the *cso* operon, but surprisingly, without a neighboring α-*mcdA* gene. These orphaned α-McdB type 2 proteins within the *cso* operon lack the charged N terminus, which is predicted to interact with McdA based on the fact that all other McdB proteins that are encoded next to the *mcdA* gene possess this charged N terminus (Fig. 2B). Also, as mentioned previously, ParA family ATPases typically have their partner protein encoded immediately downstream, and in the same operon, of the cognate *parA* gene. Several ParA partner proteins use their charged N terminus for interaction with their cognate ParA to stimulate its ATPase activity (88). It remains to be determined if the charged N terminus of McdB proteins is responsible for interaction with McdA. Also to be determined is the functional requirement of two distinct α-McdB proteins, one that presumably interacts with α-McdA and another that does not.

Regardless of McdB type, all share five core features: (i) intrinsically disordered regions (IDRs) that greatly vary in length, (ii) repetitive and biased amino acid compositions, (iii) low hydrophobicity, (iv) extreme multivalency, and (v) an invariant C-terminal tryptophan residue. Most striking is the intrinsic disorder across all identified McdB proteins (Fig. 2C). Most β-McdB proteins possess ∼50% disorder, consistent with the predicted presence of a structured coiled-coil region, while most α-McdB proteins are predicted to be completely disordered (Fig. 2B and C). These shared features of all McdB proteins are sequence hallmarks of proteins that can under liquid-liquid phase separation (LLPS) (Fig. 2D), a phenomenon that has also been recently observed with the core components of the carboxysome itself (46, 47). In the following sections, we discuss how LLPS may be involved in both carboxysome assembly and homeostasis by the McdAB system.

## THE ROLE OF LIQUID-LIQUID PHASE SEPARATION IN CARBOXYSOME ASSEMBLY

For almost 50 years, the carboxysome, BMCs in general, and the algal equivalent of the carboxysome called the pyrenoid, have all largely been viewed as paracrystalline in nature as observed by electron micrographs (63, 82, 111–116). However, recent *in vivo* fluorescence microscopy in living cells has provided compelling evidence that carboxysome homeostasis is a highly dynamic process, immediately responsive and adaptable to environmental change, including changes in growth temperature (80), CO_2_ concentration (78), light intensity (78, 79), and wavelength (81). It is not intuitively obvious how a crystalline carboxysome can dynamically and reversibly tune its copy number, size, composition, and selective permeability. Several recent landmark studies now show that the internal components of the carboxysome (both α and β) and the algal pyrenoid all share liquid-like properties and potentially form via LLPS (43, 46, 47, 117, 118), a paradigm shift in our understanding of all facets of BMC biology.

LLPS refers to the ability of macromolecules to demix into a dilute phase and a dense phase, called a “condensate” (Fig. 2D). These two phases can coexist as liquids, or the condensate can further transition into more ordered gels and solids depending on solution conditions (i.e., protein concentration, crowding, osmolarity, pH, salt type and concentration, and temperature) (119). Proteins across all domains of life are emerging with the shared ability to form membraneless organelles via the process of LLPS. Membraneless organelles have known roles in the subcellular organization of eukaryotic cells, but the study of this method of compartmentalization in bacteria is in its infancy (12). Currently, little is known about the role of LLPS in BMC assembly, homeostasis, and function or how liquid-like organelles in general are spatially regulated in bacteria.

Core components of both α- and β-carboxysomes form liquid droplets *in vitro*. For β-carboxysomes, Rubisco forms droplets with the intrinsically disordered protein CcmM (46), and for α-carboxysomes, Rubisco forms droplets with the intrinsically disordered protein CsoS2 (47). Moreover, cryo-electron microscopy (cryoET) of the β-carboxysome core finds that the Rubisco-CcmM matrix resembles the liquid-like core of the algal pyrenoid, where Rubisco condensates form with the intrinsically disordered protein EPYC1 (38, 116). Time-lapse fluorescence microscopy has shown that β-carboxysome assembly occurs from the inside-out, starting with the coalescence of a Rubisco-CcmM “procarboxysome,” which is then encapsulated by shell proteins (63, 120). The assembly pathway for α-carboxysomes is less clear, but the fact that α-shell proteins can assemble “ghost” carboxysomes, devoid of core proteins, suggests that inside-out assembly is not obligatory for α-carboxysomes (82, 87). Consistently, a recent study engineered α-carboxysome shells (∼100- nm diameter) devoid of core proteins (121). These shells were used as nanoreactors to recruit heterologous enzymes for diverse catalytic reactions.

The *in vitro* studies detailed above suggest the procarboxysome is a liquid-like condensate, but liquidity has yet to be directly established *in vivo*. Despite this, many *in vivo* behaviors of fluorescent carboxysomes in living cells correlate with a liquid-like nature, including dynamic tunability to environmental change, and the ability to reversibly grow, shrink, fuse, and bud. While compelling evidence is mounting that carboxysome cores can be considered condensates, it is important to note that the carboxysome is not a typical “membraneless organelle.” Carboxysomes have a selectively permeable protein shell and are therefore not membraneless. The coupling of core condensation with shell encapsulation provides bacteria with a powerful strategy to control BMC size, composition, and selective permeability.

It is attractive to speculate that shell encapsulation influences the material state of the carboxysome core. For example, the degree of shell encapsulation could tune the viscosity of the enzymatic core, thus also possibly tuning the enzymatic activity of Rubisco. Alternatively, the carbon-fixing activity itself could be modulating carboxysome fluidity. Indeed, it has been proposed that metabolic activity can fluidize the bacterial cytoplasm from a glass-like state to a liquid state (122). Heterogeneity in Rubisco packing and carboxysome morphology, from defined icosahedral to amorphous blob, has been shown *in vivo* (14, 123), but whether carboxysomes undergo reversible shifts between crystalline and liquid states, and whether these shifts correlate with changes in carbon-fixation efficiency, remains to be elucidated.

## THE ROLE OF LIQUID-LIQUID PHASE SEPARATION IN CARBOXYSOME POSITIONING

In addition to carboxysome core components displaying liquid-like behaviors, McdB proteins also undergo LLPS *in vitro* (41, 44), the first example of a ParA family partner protein exhibiting this behavior. How McdB associates with carboxysomes remains unclear, but LLPS activity has been proposed to be involved in McdB recruitment to carboxysomes (44). Specifically, *S. elongatus* McdB droplet formation *in vitro* is pH dependent (Fig. 2E). This observation is informative since it has recently been proposed that Rubisco proton production drives the elevation of CO_2_ within carboxysomes, which would generate a pH gradient between the cytoplasm and the carboxysome lumen (53, 124–126). Indeed, while the cytosolic pH of *S. elongatus* is ∼8.5 in light-acclimated cells, metabolically active carboxysomes are predicted to be relatively acidic (pH 6 to 7) (127). An acidic carboxysome would increase the maximum carboxylation rate of Rubisco and reduce the amount of HCO_3_^−^ uptake required to saturate Rubisco (127). *In vitro*, McdB is soluble at a pH of ≥8 and forms droplets at a pH of ≤7.5 (Fig. 2E), suggesting McdB would remain soluble in the *S. elongatus* cytoplasm and would undergo LLPS on metabolically active carboxysomes (Fig. 2F) (44). Consistently, fluorescent McdB is completely diffuse in the cytoplasm of light-acclimated *S. elongatus* cells lacking carboxysomes (40). In dark-acclimated cells, the cytosolic pH of *S. elongatus* drops to ∼7.3 (127). It remains to be determined if McdB condensation *in vivo* can be regulated by day-night cycles, but this form of LLPS regulation has recently been found to occur for a large subset of the *S. elongatus* proteome (128). In this study, fluorescent-labeled proteins formed puncta at night, which then reversibly solubilized into the cytoplasm in the morning. The circadian clock regulates the formation and dissolution of these puncta, and the formation of condensates reflected the metabolic status of the cell. Similarly, the circadian clock may regulate McdB condensation on carboxysomes, and this activity may reflect the metabolic status of the carboxysome itself. In line with this proposal, a recent study explored the diurnal regulation of carboxysomes in *S. elongatus* and found that, in the dark, cells have fewer carboxysomes, and a greater fraction were mislocalized to the cell poles (129). It is possible that this diurnal control of carboxysome positioning is mediated by the McdAB system.

We propose that the McdAB system uses pH as a read-out for the metabolic status of individual carboxysomes (Fig. 2F) (44). Carboxysomes that are efficiently fixing carbon would have a sufficiently low pH for McdB recruitment via LLPS and would therefore be recognized as cargo by McdA for positioning on the nucleoid. Carboxysomes that are metabolically inert, on the other hand, would not have the low pH required for McdB recruitment. Carboxysomes lacking McdB are not positioned by McdA and become nucleoid excluded to the cell poles (40). Consistently, inactive carboxysomes have recently been shown to move to the poles of a cyanobacterial cell immediately prior to their degradation (108). It is therefore attractive to speculate that the McdAB system can sense which carboxysomes are active and require positioning and which are inactive and should be targeted for degradation.

## MOVING FORWARD WITH MCDAB SYSTEMS AND CARBOXYSOME POSITIONING

Recent studies of the McdAB system are beginning to unveil the general principles of BMC spatial organization, which also has implications for understanding ParA-based organization of other mesoscale assemblies across the bacterial world. If carboxysome cores are indeed liquid-like, this makes the McdAB system one of the first examples of an ATP-driven organizing system for liquid-like organelles in bacteria. It remains to be determined how the McdAB system regulates the formation, function, and organization of carboxysomes. It is possible that the McdAB system governs carboxysome homeostasis via active regulation of its LLPS activity, but how McdB connects to the carboxysome shell and its liquid-like core remains unknown. It also remains to be determined what factors regulate the LLPS activity of McdB, what regions of McdB are required for LLPS, what the material properties of McdB droplets are, and how these material properties influence carboxysome function. McdB-like proteins are found in other BMC-containing bacteria (41, 44) yet remain a novel and uncharacterized family of proteins. We hypothesize that McdA gradients on the nucleoid generate pulling forces on McdB-fluidized BMCs to promote their fission, partition, and distribution in the cell. An exciting future direction is to determine how the ATPase activity of McdA actively segregates and distributes McdB-bound, and potentially liquid-like, carboxysomes.

Cyanobacteria possess a circadian clock that precisely operates on the 24-h rotational period of the earth, which allows cells to anticipate, adapt, and respond to daily light cycles by translating environmental cues into changes in gene expression (130). In *S. elongatus*, oscillatory patterns of gene expression are driven by phosphorylation of the master output transcriptional regulator protein RpaA. Phosphorylated RpaA binds ∼170 promoters of the *S. elongatus* chromosome (131); one site is the promoter for the *mcdAB* operon. Therefore, it will be interesting to explore the role of McdB LLPS activity at carboxysomes and how circadian rhythms and light-dark conditions influence McdAB expression, dynamics, and function. Moreover, the nucleoid upon which McdA oscillates undergoes compaction and relaxation over circadian cycles (132–134). This could partially explain why some cells have linearly arranged carboxysomes, while other cells have carboxysomes that are hexagonally packed. How changes in nucleoid compaction influence McdA dynamics and subsequent carboxysome positioning remains an outstanding question.

Unlike cyanobacteria, which perform oxygenic photosynthesis, the metabolisms of α-carboxysome-containing proteobacteria greatly vary. Despite this, α-McdAB systems are present in nitrite, ammonia, and iron utilizers, as well as in sulfur-oxidizing chemoautotrophs and purple sulfur bacteria, which perform anoxygenic photosynthesis (41). β-carboxysome homeostasis responds to changes in temperature, CO_2_ levels, and light during cell growth (78–81). Given the diversity of metabolic substrates utilized among α-carboxysome-containing proteobacteria, it is possible that α-carboxysome homeostasis is also regulated by a variety of external cues, such as nutrient availability.

Finally, heterologous expression of α- or β-carboxysomes to endow heterotrophic bacteria with carbon-fixing activity (135, 136), or to turbo-charge carbon fixation in plant chloroplasts (137–143), has been a long-standing biotechnological goal. While functional carboxysomes have been assembled, the carboxysomes coalesce to form massive aggregates that are nucleoid-excluded in bacterial cells (135, 136) or randomly located within spacious regions of chloroplasts (143). However, a very recent study coexpressed the α-McdAB system with α-carboxysome components of *H. neapolitanus* in Escherichia coli cells (144). Consistent with the idea that McdA and McdB are both necessary and sufficient for distributing carboxysomes, electron-micrographs show α-carboxysomes distributed across the cell length and along the E. coli nucleoid. Given the penetrance of McdAB systems across most organisms with carboxysomes, the importance of the McdAB system for carboxysome homeostasis and function cannot be understated. Therefore, we recommend that future efforts in introducing carboxysomes into heterologous hosts also include their cognate McdAB system.

## SEVERAL BMC OPERONS ENCODE FOR PUTATIVE POSITIONING SYSTEMS

McdAB is the first example of an ATP-driven system capable of spatially organizing a BMC and represents a tractable model for understanding active homeostasis mechanisms governing bacterial organelles. We anticipate that a deeper understanding of carboxysome homeostasis, including nontraditional mechanisms of assembly involving LLPS and ATP-driven organization is just on the horizon.

One outstanding question is that while *S. elongatus* and *H. neapolitanus* are rod-shaped bacteria, McdAB systems have been identified in cells of diverse morphologies (44). For example, several cyanobacteria with McdAB systems are spherical. Mathematical modeling of the Brownian-ratchet mechanism suggests that carboxysome positioning by the McdAB system is indeed influenced by cellular geometry but still operates within spherical cells to optimally space carboxysomes from one another (40). Experimentally addressing how the McdAB system behaves within these unique cellular geometries is of profound interest.

Another outstanding question is whether the McdAB system is restricted to carboxysomes. Several BMC operons encode putative McdAB systems (18, 41, 44). These McdA- and McdB-like proteins are encoded within or neighboring the BMC operon. In cases where McdB-like sequences are observed, all possess a C-terminal aromatic residue, a feature that is invariant across all carboxysome-associated McdB proteins we have identified to date (Fig. 2B). This amino acid is intriguing because many proteins involved in the assembly of viral or phage capsids also encode an aromatic residue (tryptophan) at their C terminus (145–150). Given the capsid-like icosahedral structure of BMCs, it is attractive to speculate that C-terminal aromatic residues play a role in McdB association with their cognate BMC. Elucidating the carboxysome homeostasis mechanisms provided by the McdAB system will unveil shared principles of organization for BMCs encoding these putative systems.

Bioinformatic analyses suggest that active BMC organization is not restricted to McdAB-like systems (18). Actin-like proteins (PF06723 and PF11104) are encoded in a number of BMC operons. Also, PduV (PF10662), the Ras-like GTPase suggested to play a role in the spatial positioning of the PDU BMC (35), has homologs encoded in most PDU, EUT, and glycyl radical enzyme-associated microcompartment (GRM) loci (18). The prevalence of these NTPases neighboring BMC operons suggests that subcellular organization is of general importance to BMC function.

Understanding the mechanisms associated with BMC organization has human health implications. In the human gut microbiome, several metabolosomes have been shown to be involved in metabolism and bacterial pathogenesis (9). Therefore, BMCs and their unstudied positioning systems are possible targets for the design of narrow-spectrum antibiotics. From a synthetic biology perspective, carboxysome bioengineering and the design of synthetic BMCs for medical and biotechnological applications are areas of intense research (135–140), yet realizing their potential relies on determining the key principles of BMC assembly, organization, and homeostasis in the cell.
